# Oxidoreductases that Act as Conditional Virulence Suppressors in *Salmonella enterica* Serovar Typhimurium

**DOI:** 10.1371/journal.pone.0064948

**Published:** 2013-06-04

**Authors:** Naeem Anwar, Xiao Hui Sem, Mikael Rhen

**Affiliations:** 1 Department of Microbiology, Tumor and Cell Biology, Karolinska Institutet, Stockholm, Sweden; 2 Singapore Immunology Network, Agency for Science, Technology and Research, Singapore, Republic of Singapore; Indian Institute of Science, India

## Abstract

In *Salmonella enterica* serovar Typhimurium, oxidoreductases of the thioredoxin superfamily contribute to bacterial invasiveness, intracellular replication and to the virulence in BALB/c mice as well as in the soil nematode *Caenorhabditis elegans.* The *scsABCD* gene cluster, present in many but not all enteric bacteria, codes for four putative oxidoreductases of the thioredoxin superfamily. Here we have analyzed the potential role of the *scs* genes in oxidative stress tolerance and virulence in *S.* Typhimurium. An *scsABCD* deletion mutant showed moderate sensitization to the redox-active transition metal ion copper and increased protein carbonylation upon exposure to hydrogen peroxide. Still, the *scsABCD* mutant was not significantly affected for invasiveness or intracellular replication in respectively cultured epithelial or macrophage-like cells. However, we noted a significant copper chloride sensitivity of SPI1 T3SS mediated invasiveness that strongly depended on the presence of the *scs* genes. The *scsABCD* deletion mutant was not attenuated in animal infection models. In contrast, the mutant showed a moderate increase in its competitive index upon intraperitoneal challenge and enhanced invasiveness in small intestinal ileal loops of BALB/c mice. Moreover, deletion of the *scsABCD* genes restored the invasiveness of a *trxA* mutant in epithelial cells and its virulence in *C. elegans.* Our findings thus demonstrate that the *scs* gene cluster conditionally affects virulence and underscore the complex interactions between oxidoreductases of the thioredoxin superfamily in maintaining host adaptation of *S*. Typhimurium.

## Introduction

*Salmonella enetrica* serovar Typhimurium (*S*. Typhimurium) is a facultative intracellular enteric pathogen that traditionally has been used as a model organism for studying typhoid fever caused by the human adapted serovar Typhi [1], [2]. In the murine model for typhoid fever, *S*. Typhimurium invades the intestinal epithelium after *per oral* challenge. Invasion is followed by dissemination and intracellular bacterial replication in phagocytic cells of the liver and spleen.

Virulence of *S*. Typhimurium in mammalian cell culture and in mice, as well as in alternative host infection models, strongly relies on horizontally acquired DNA regions termed as *Salmonella* pathogenicity islands (SPI:s) [3]. Two of the SPI:s, SPI1 and SPI2, code for protein type III secretion systems (T3SS) that translocate bacterial effector proteins into host cells. The effector proteins act to manipulate central host cell functions, such as actin polymerization, vesicular trafficking, and signal transduction [4], [5], [6]. In mice, SPI1 and SPI2 enable the bacteria to transcytose the intestinal epithelial barrier, and disseminate to replicate in macrophages of the liver and spleen respectively [7], [8]. Moreover, SPI1 and SPI2 T3SS also modulate pro-inflammatory responses of the host during *Salmonella* infection [9], [10], [11].

Reactive oxygen species (ROS) generated by *Salmonella*-induced inflammatory responses most evidently contribute to the clearance and pathogenesis of salmonellosis. Mice (*phox* −/−) lacking a functional phagocyte oxidase rapidly succumb upon infection doses well controlled by wild type littermates [12]. Furthermore, individuals suffering from chronic granulomatous disease, a condition marked by a defective oxidative phagocyte response, show an increased prevalence of extraintestinal infections caused by non-typhoidal serovariants of *Salmonella* [13].

The genome sequence of *S. enterica* reveals a number of enzymes that potentially provide protection against ROS, such as H_2_O_2_. These enzymes include super oxide mutases, catalases, thiol peroxidases and methionine sulphoxide reductases [14], [15], [16], [17]. In addition, oxidoreductases of the thioredoxin superfamily contribute to the assembly or activities of many bacterial virulence factors. SPI2 activity in part relies on the periplasmic DsbA and SrgA proteins, which both belong to the thioredoxin superfamily of oxidoreductases [18]. SrgA is in fact coded for by a plasmid-carried fimbrial operon *pef*, and moreover is required for the assembly of Pef fimbriae [19]. The activity of SPI2 also depends on the cytoplasmic thioredoxin 1 (TrxA, *trxA*), whereby *trxA* mutants are severely attenuated for replication in macrophage-like cells and in mice [20], [21]. TrxA additionally mediates a redox-associated lethality caused by *S*. Typhimurium in an infection model based on the soil nematode *C. elegans* [22].

**Figure 1:**
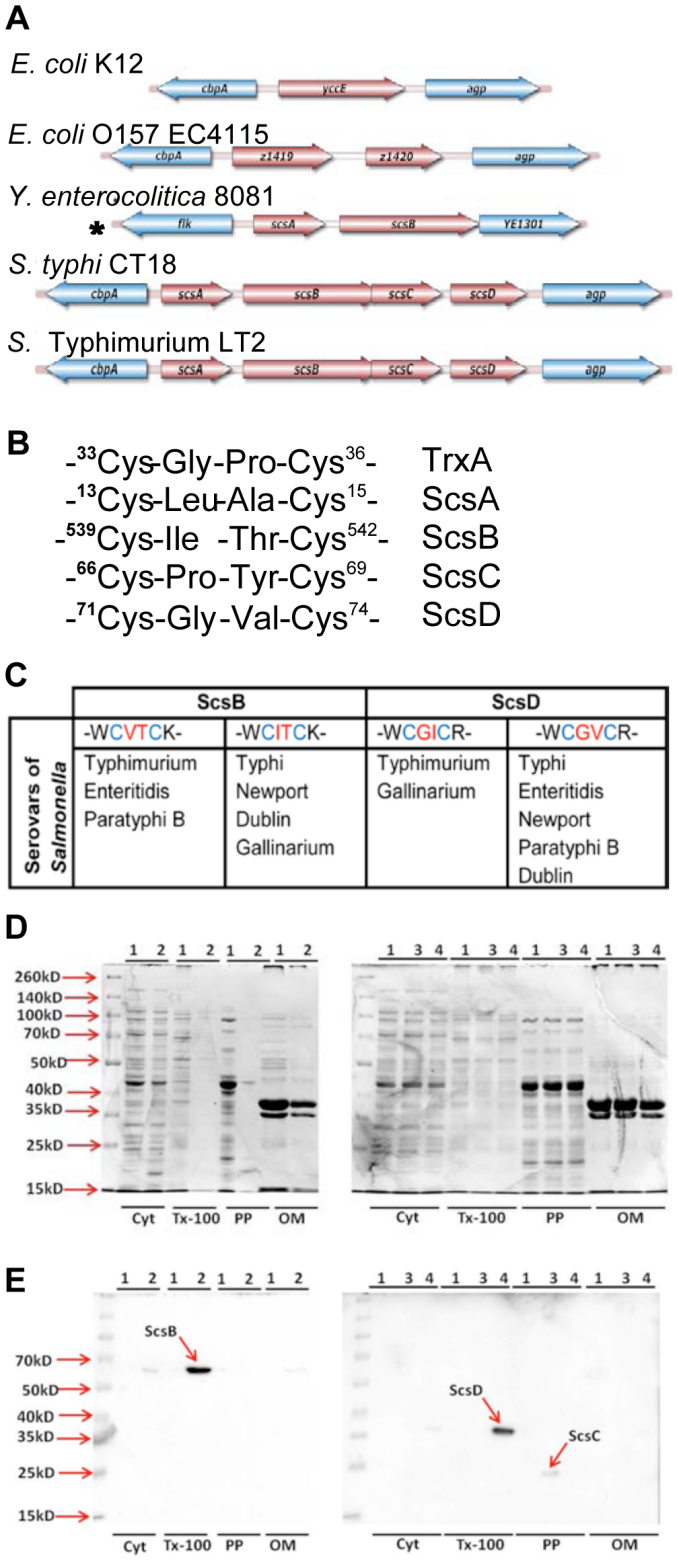
In silico analyses and localization of the ScsABCD proteins. A) Illustrates the cbpA-agp genomic region in selected strains of Eschericia coli (E. coli), Yersinia enterocolitica (Y. enterocolitica) and Salmonella enterica (S. enterica); * No cbpA or agp present. B) and C) detail and compare the Cys-X-X-Cys motives of Scs proteins with the prototype TrxA and their variability in predicted SccB and ScsD proteins. D) shows Coomassie blue stained 12%SDS-polyacrylamide gels presenting sub-cellular fractionation of the proteins E) presents immunoblot with anti-His to show the localization of Scs proteins in different subcellular fractions. In (D) and (E), cyt = cytoplasmic fraction; Tx-100 =  Triton X-100 soluble fraction; PP = periplasmic fraction and OM = Tx-100 insoluble integral outer membrane fraction. 1 =  WT/pET32a; 2 =  scsB clone (pNA15), 3 =  scsC clone (pNA16) and 4 =  scsD clone (pNA17).

Many of the oxidoreductases such as TrxA, DsbA and thiol peroxidase Tpx implicated in virulence of *S.* Typhimurium and in redox tolerance of *E. coli*, are highly conserved within enterobacteria [15], [18], [23]. In contrast to laboratory *E. coli* strains, *S*. Typhimurium includes the *scsABCD* gene cluster that encodes four proteins each with a *Cys-X-X-Cys* motif characteristic for the thioredoxin superfamily [24] (Figs. 1A and 1B). When cloned into *E. coli*, the *scs* genes restore copper tolerance of selected copper-sensitive mutants [24]. However, the actual role of the *scs* genes for redox tolerance in *S*. Typhimurium and their contribution to virulence, if any, has remained enigmatic. Here we demonstrate that a *S*. Typhimurium *scsABCD* deletion mutant shows moderate sensitization to copper chloride and, surprisingly, a conditional enhanced invasiveness in epithelial cells and virulence in *C. elegans*.

## Results

### The *scsABCD* Gene Cluster

The predicted primary sequences of the *S*. Typhimurium ScsABCD proteins were presented in 1997 [24]. Since, a number of new genome sequences and protein prediction algorithms have been annotated, whereby homologues of Scs proteins have been identified in a number of diverse bacterial species [25]. Hence, we revisited the *S. enterica scsABCD* genes and protein predictions for the ScsABCD protein sequences in terms of *in silico* analyses using more recent public databases.

In the genome sequence of *S.* Typhimurium LT2, the *scsABCD* genes position between *cbpA* and *agp* genes [26]. The *scs* genes are contained in two transcriptional units read from the same DNA strand [24]; one consistsing of *scsA* followed by a second including the *scsBCD* genes. In contrast, *E. coli* K-12 and many other members of *Enterobacteriacae* and related organisms lack *scs* gene sequences, or contain only selected *scs* genes positioned between *cbpA* and *agp* genes (Fig.1A).

Each of the *scs* genes codes for a predicted protein with a single *Cys-X-X-Cys* motif and an enriched containment of hydrophobic amino acids between the *Cys* residues (Fig. 1B). Such a motif fits the hallmark of the catalytic site of the thioredoxin/glutaredoxin family of oxidoreductases. The *Cys-X-X-Cys* motifs for ScsA and ScsC are conserved in all annotated *S. enterica* genome sequences, whereas for ScsB and ScsD either one of the two residues between the cysteines is variable (Fig. 1C) potentially leading to alterations in the redox potential of the proteins [27], [28].

According to signal sequence predictions for the *S*. Typhimurium Scs proteins, only ScsC would contain a classical signal sequence. This would also fit with its suggested role as a periplasmic disulphide isomerase in *Caulobacter cresentus* [25]. ScsA and ScsB both contain a putative lipobox at their N-termini that predict the proteins to be outer membrane lipoproteins. However, for ScsA any processing at the predicted lipobox sequence would delete the *Cys-X-X-Cys* motif, while for ScsB the putative lipobox sequence positions at only 13 residues from the initiation methionine. Still, ScsB as well as ScsD were predicted to be integral membrane proteins.

### Expression and Localization of Recombinant Scs Proteins

To get indications for the actual localization of the Scs proteins we first cloned them individually in the pBAD30 vector that allows for L-arabinose assisted inductions of recombinant proteins in *S*. Typhimurium. Each *scs* gene was amplified individually including its putative ribosome binding site at the 5′-end, and coding for C-terminal 6xHis tag. Attempts to express the proteins in *S*. Typhimrium only revealed signal for ScsC upon immunobloting for the His-tag at the expected mass position (data not shown). However, detection of plain ScsA, ScsB or ScsD proteins in *S.* Typhimurium appeared difficult.

One possibility could be extreme sensitivity to proteolytic degradation, or suboptimal induction of ScsA, ScsB or ScsD in *S*. Typhimurium from our recombinant plasmid constructs. In a next attempt we tried to express all Scs proteins individually in *E. coli* BL21 strain, deficient in the cytoplasmic Lon protease and expressing the phage T7 RNA polymerase, using the T7 promoter-based expression vector pET32a. The recombinant BL21 *E.coli* strain was next fractionated into a detergent insoluble cell wall fraction (representing integral outer membrane proteins), detergent soluble cell wall fraction, periplasmic protein fraction and cytosolic fraction. The fractions were separated on SDS-PAGE gels (Fig. 1D) and used for immunobloting of the His-tag. In this scenario, a band corresponding to calculated molecular mass of ScsC-6xHis clearly appeared enriched in the periplasmic fraction at 23-kDa as was detected in *S.* Typhimurium (Fig. 1E). This would be consistent with the predicted periplasmic localization of the protein.

Similarly, cloning of *scsB* with His-tag coding region into pET32a allowed for detection of a 67-kDa band in the detergent soluble fraction (Fig. 1E). This would be consistent with the predicted localization of ScsB within the inner membrane, the localization predictions recently presented for the ScsB superfamily of redox transporters [25] and with the predicted apparent mass of the fusion protein.

In selected cases, introduction of an N-terminal thioredoxin 1 tag results in stabilization of recombinant proteins. Such a strategy was considered inappropriate for ScsA as a concomitant fusion protein would be severely affected for any subsequent signal peptide processing. However, predicted to be devoid of the signal sequence, for ScsD we generated a translational thioredoxin-ScsD-His-tag fusion protein as coded for by pET32a. Upon induction in *E. coli* BL21, we noted an additional protein band of 36-kDa molecular mass in the detergent soluble fraction in Coomassie stained protein gel (data not shown). This matched the predicted size of a TrxA-ScsD-His fusion protein. A band at the same position was detected upon immunoblotting for the His-tag (Fig 1E). When excised from the gel, and subjected to mass-spectrometric analyses, it revealed both thioredoxin and ScsD peptides. Admittedly, this fusion protein clearly is artificial, but the analyses would be in line with an assumed cytoplasmic membrane association of ScsD, and assuming that ScsD contains a strong enough internal topological membrane insertion domain(s).

### Construction of *scs* Deletion Mutants

In order to study any functional aspect of the *scs* genes in *S*. Typhimurium, we set out to delete each reading frame using recombinase-assisted site-directed mutagenesis [29]. For *scsA*, the deletion comprised the entire reading frame, but not extending into the intergenic region between *scsA* and *scsB* (for primer details, see Table S2). The open reading frames (ORF) of *scsB* and *scsC* partially overlap [24]. Therefore the deletions of the individual *scsB*, *scsC* and *scsD* genes were designed not to interfere with the ORFs of the rest. We also created a strain construct, a Δ*scsABCD* quadruple deletion mutant, lacking all the four *scs* genes from the 5′-end of *scsA* to the 3′-end of *scsD* ORFs. Finally, to exclude any polar effects caused by the resistance cassette used for tagging the mutations, the cassettes were removed from all deletion mutants.

### The *scsBCD* Genes Equally Contribute to Copper Chloride Tolerance in *S*. Typhimurium

Copper chloride (CuCl_2_) sensitivity in *E. coli* mutants is reversed when provided with the cloned *scsABCD* genes from *S*. Typhimurium [24]. Therefore we started to probe for the contribution of *scs* genes to the intrinsic copper tolerance in *S*. Typhimurium using the deletion mutants defined above.

**Figure 2:**
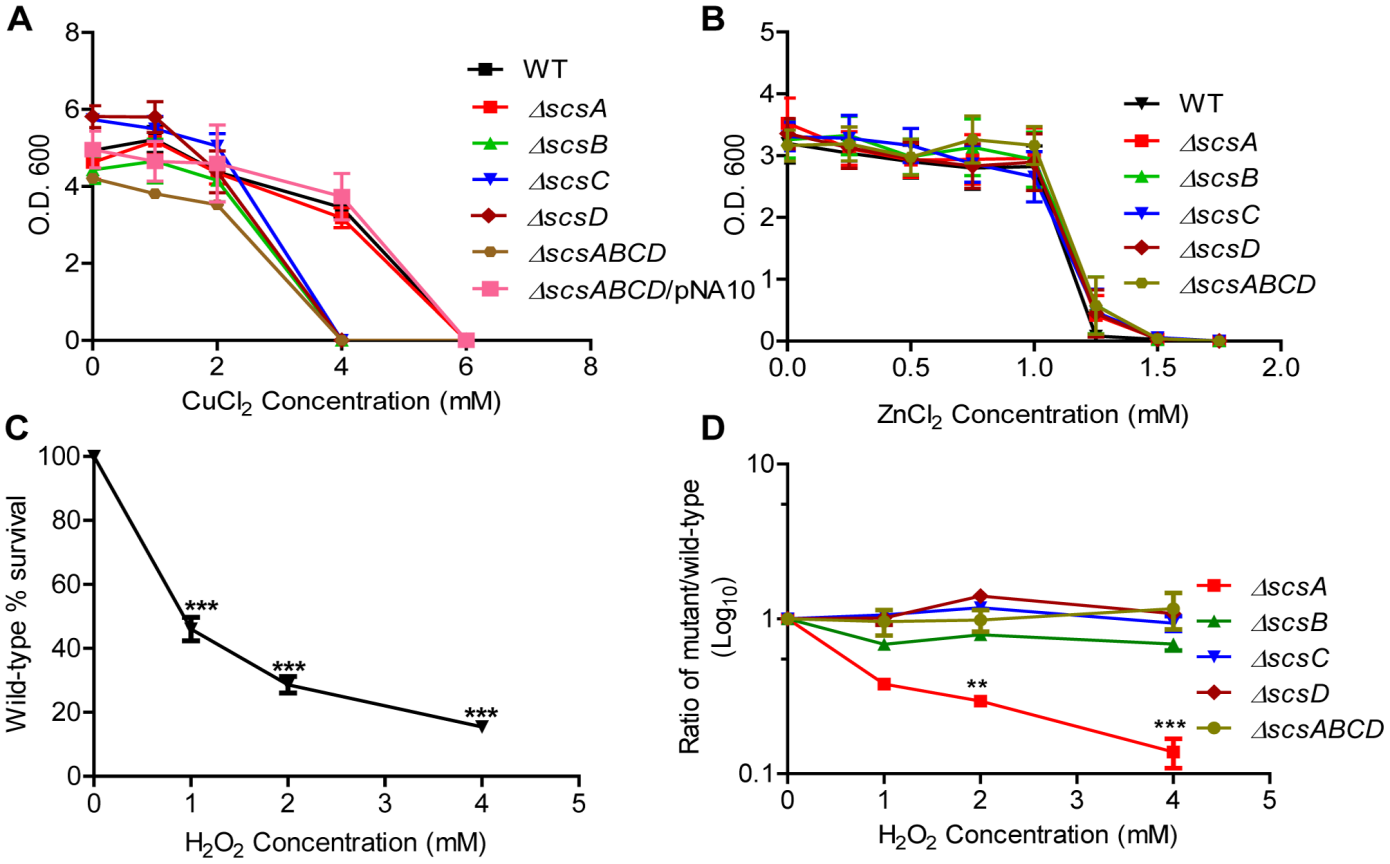
Sensitivity to copper chloride, zinc chloride and hydrogen peroxide. A) Optical densities of overnight wild type S. Typhimurium and different scs mutants grown in the presence of CuCl_2_. This sensitivity is reversed by trans-complementation using the cloned *scsABCD* genes on plasmid pNA10 for Δ*scsABCD* mutant. B) shows equal sensitization to ZnCl_2_ for wild type S. Typhimurium and its Δscs mutants. C) Loss in viable count of the wild type S. Typhimurium strain after exposure to H_2_O_2_. Values are given as percentage relative to a hydrogen peroxide deficient control. *** = p≤0.001 D) Relative indices of viable counts of different scs knockouts compared to wild type reveals sensitization only for the ΔscsA mutant. ** = p≤0.01, *** = p≤0.001. Error bars indicate standard error of the mean.

The Δ*scsA* mutant did not reveal CuCl_2_ sensitivity while deleting either the *scsB*, *scsC* or *scsD* gene resulted in a moderate but equal and highly reproducible sensitization to CuCl_2_ (Fig. 2A). Similarly, the Δ*scsABCD* quadruple deletion mutant showed the same sensitization as the individual Δ*scsB,* Δ*scsC* and Δ*scsD* mutants (Fig. 2A).

Attempts to complement individual Δ*scs* mutants with a cloned corresponding gene failed (data not shown). However, all copper sensitive single Δ*scs* mutants as well the quadruple deletion mutant, were fully complemented for CuCl_2_ tolerance by the *scsABCD* genes cloned in cloning vector pSU41 (pNA10) (Fig. 2A and data not shown). None of the mutants revealed increased sensitization to another antibacterial transition metal in the form of zinc chloride (Fig. 2B).

### The *scsABCD* Genes Protect against Protein Carbonylation

As CuCl_2_ mediates cysteine disulphide bond formation and potentially promotes generation of ROS, we next tested for any sensitization of the *scs* mutants to hydrogen peroxide (H_2_O_2_). In doing this we neither observed H_2_O_2_ sensitization for the Δ*scsB,* Δ*scsC and* Δ*scsD* single mutants nor for the Δ*scsABCD* quadruple mutant (Fig. 2D). Notwithstanding the fact, the Δ*scsA* single mutant showed increased sensitivity to H_2_O_2_ (Fig. 2D).

**Figure 3:**
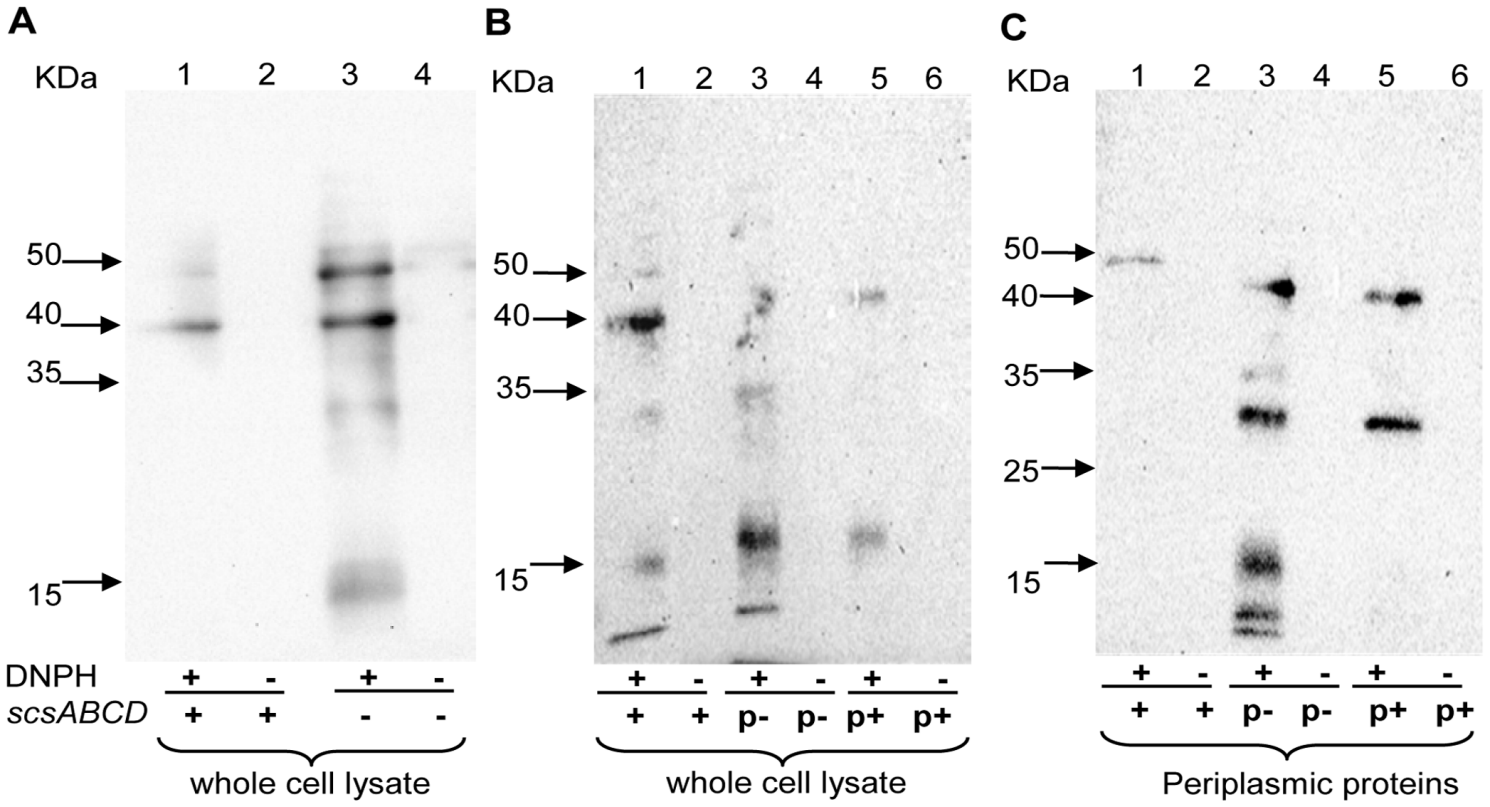
Carbonylated protein profiles after exposure to hydrogen peroxide. Protein carbonylation was detected by immunoblotting after coupling to dinitrophenol and protein separation on 12% SDS-PAGE gels. A) Whole cell lysate obtained from the Δ*scsABCD* mutant reveals a higher concentration of carbonylated proteins after exposure to 0.75 mM hydrogen peroxide (B). Trans-complementation with cloned *scsABCD* (pNA10) reduces the level of protein carbonylation in the whole cell fraction, as well as in the periplasmic fraction (C). DNPH = Dinitrophenyl hydrazine (+) = presence (−) = absence. p+ indicates the trans-complementing *scsABCD* genes in pNA10. p- indicates the vector control pSU41.

Apart from mediating disulphide bond formation, ROS also results in protein carbonylation in *S*. Typhimurium [17]. Thus, we assayed protein carbonylation in cultures of *S*. Typhimurium exposed to 0.75 mM H_2_O_2_ to detect any discernible differences_._ To exclude any confounding effects by the Δ*scsA* single mutant, we continued using the Δ*scsABCD* mutant only. The rationale for this was that this mutant lacked all the *scs* genes yet did not reveal sensitization to H_2_O_2._ In this experiment we detected a more pronounced accumulation of carbonylated proteins in the Δ*scsABCD* mutant (Fig. 3A), notably in the periplasmic fraction (Fig. 3C). Expressing the cloned *scsABCD* genes from pNA10 in the quadruple mutant reduced the H_2_O_2_-mediated protein carbonylation (Fig. 3B). That rather few proteins became increasingly carbonylated in the Δ*scsABCD* mutant could explain why this *scs* mutant did not display increased loss of viability upon exposure to H_2_O_2_. Still, these series of experiments strongly implicated that the Scs proteins are involved in balancing periplasmic oxidative stress in *S*. Typhimurium.

### *S*. Typhimurium Still Relies on *trxA* for Copper Chloride Tolerance

**Figure 4:**
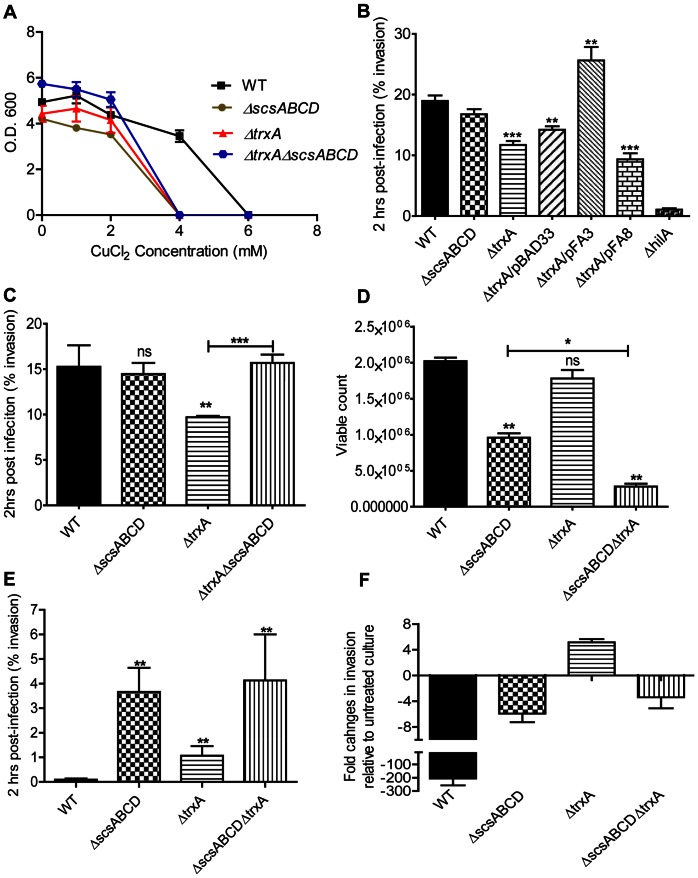
Sensitivity to copper chloride and invasion in MDCK epithelial cell line, with or without copper chloride stress. A) Optical density of overnight wild type S. Typhimurium, Δ*scsABCD* and different Δ*trxA* mutants grown in the presence of CuCl_2_. B) The compromised invasion of a Δ*trxA* deletion mutant can be trans-complemented with cloned *trxA* (pFA3) but not with a plasmid coding for catalytically inactive TrxA (pFA8). C) The *scsABCD* deletion acts as a suppressor mutation for the decreased invasion of the Δ*trxA* mutant. D) and E) The viability of Δ*scsABCD* and Δ*trxA* under CuCl_2_ stress (3 mM) is compromised, yet the invasiveness is highly enhanced at this concentration. F) shows fold changes in invasion of 3 mM CuCl_2_ treated culture relative to the untreated culture. ns = non-significant; * = p≤0.05; ** = p≤0.01; *** = p≤0.001.The error bars indicate standard error of the mean.

TrxA also contributes to copper tolerance in *E. coli* [30]. We thereby set out to test whether the presence of *scsABCD* genes made *trxA* dispensable for CuCl_2_ tolerance in *S*. Typhimurium. We noted a decreased CuCl_2_ tolerance of a *S*. Typhimurium Δ*trxA* mutant that in magnitude equaled to that of the *ΔscsABCD* mutant (Fig. 4A). *S*. Typhimurium lacking *trxA* as well as the *scsABCD* genes did not show any further sensitization to CuCl_2_ (Fig. 4A).

### Redox Sensitivity and Dependency on *scsABCD* Genes for *in vitro* Invasiveness of *S*. Typhimurium

Full invasiveness of *S*. Typhimurium in mammalian cell cultures relies on *trxA* [20]. As *trxA* and the *scsABCD* mutants revealed an equal sensitization to CuCl_2_, we set testing that to what extent the invasiveness was affected in our mutants, and whether invasiveness would be influenced by CuCl_2_. For this, the cultures were grown to induce SPI1 expression in the absence or presence of CuCl_2_ for 4 hours, and subsequently exposed to MDCK epithelial cells without CuCl_2_ for one hour.

When cultures were propagated in the absence of CuCl_2_, we corroborated the decreased invasion reported for a Δ*trxA* mutant [20] (Fig. 4B). This decrease in invasiveness was retained when the mutant was provided with the cloning vector pBAD33 or when provided with a pBAD33-derivative coding for a catalytically inactive TrxA. When complemented with the wild type *trxA* in pBAD33, invasiveness was enhanced above wild type levels (Fig. 4B). These observations clearly implicate a role for TrxA in invasiveness of *S*. Typhimurium.

The Δ*scsABCD* quadruple mutant invaded as efficiently as the parental wild type strain (Fig. 4B). Surprisingly though, deleting the *scsABCD* genes from the Δ*trxA* mutant resulted in increased invasiveness (Fig. 4C).

When the cultures were grown to induce SPI1-mediated invasiveness at 3 mM of CuCl_2_ we noted a moderate decrease in colony forming units in relation to the optical density of the cultures for the Δ*scsABCD* and Δ*trxA* mutants (Fig. 4D). Still, while not reduced for viability, the invasiveness of the wild type *S*. Typhimurium strain was virtually lost upon growth under CuCl_2_ stress (Fig. 4E and 4F). In contrast, even though the Δ*trxA* mutant showed decreased invasion under ordinary growth conditions, the invasion index for the mutant became increased by pre-exposure to CuCl_2_ (Fig. 4E and 4F). Also, the relative invasiveness of both the Δ*scsABCD* and Δ*scsABCD/*Δ*trxA* mutant was much retained upon growth in the presence of CuCl_2_ (Fig. 4E and 4F). These results showed that invasiveness of *S.* Typhimurium is strongly reduced by CuCl_2_, yet that this chemical attenuation depended on the Scs and TrxA oxidoreductases.

As the invasiveness of *S.* Typhimurium in cell cultures strongly relies on SPI1 T3SS and the fact that *scsABCD* mutant retained invasiveness at CuCl_2_ stress evoked the question whether this enhanced invasion of the *scsABCD* relied on SPI1 T3SS. Therefore, we inserted a polar Tn5::*lacZY* mutation in the *scsABCD* mutant inactivating the main SPI1 transcriptional activator gene *hilA*, and another in the *prgH* gene coding for a central component of the SPI1 T3SS apparatus. When exposed to CuCl_2_, the accompanying *hilA* and *prgH* mutants failed to reveal invasiveness in the MDCK cell based assay, to an extent that approached our detection limit (data not shown). Thus, the retained invasiveness of the *scsABCD* mutant at CuCl_2_ exposure continued to be SPI1-dependent.

### The *scsABCD* and SPI1 Gene Expression

**Figure 5:**
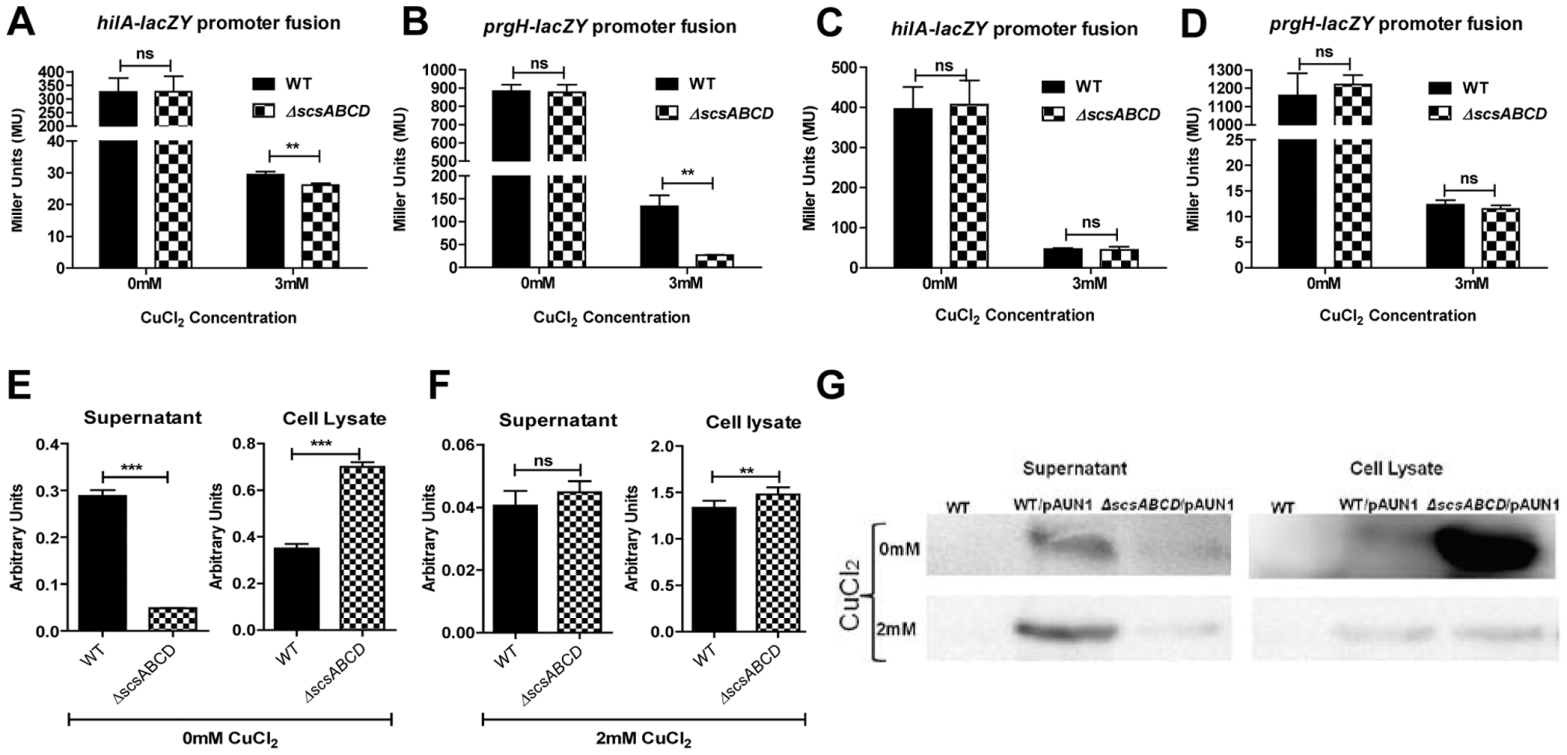
β-galactosidase and β-lactamase activities and expression measurements. A) and B) β-galactosidase activities for hilA and prgH promoter fusions from overnight LB culture are slightly reduced for *scsABCD* mutant in the presence of CuCl_2_. C) and D) β-galactosidase activities for hilA and prgH promoter fusions from 4 hrs DMEM-based invasive culture are unaffected irrespective of *scsABCD* mutation. β-lactamase activities from supernatants and cell lysates of the invasive cultures of wild type and in Δ*scsABCD* mutant bacteria in the absence (E) and presence (F) of CuCl_2_. G) β-lactamase as assayed from the same cultures by immunoblotting for β-lactamase. ns = non-significant; ** = p≤0.01; *** = p≤0.001. Error bars indicate standard error of the mean.

That the *scsABCD* mutant retained invasiveness upon CuCl_2_ stress in a SPI1-mediated manner led us to test whether the *scs* gene cluster affected SPI1 gene expression. For this we measured SPI1 gene expression using the two forth mentioned SPI1 Tn5::*lacZY* constructs. When grown over night in LB we noted clear reading of the *hilA::lacZY* and *prgH::lacZY* transcriptional fusions by assaying beta-galactosidase activities (Fig. 5A and B). Addition of CuCl_2_ to the culture prior to the inoculation resulted in an overall decreased reporter activity with a slight relative decrease in both fusions for the *scsABCD* mutant (Fig. 5A and B). However, this difference disappeared when bacteria were grown for invasiveness in cull culture medium supplemented with CuCl_2_ (Fig 5C and D).

### The *scsABCD* Genes and SPI1 Activity

To address whether *scsABCD* genes affected SPI1 activity, we followed the expression and secretion of a SPI1 effector fusion protein. For this we used plasmid pAUN1 that carries the 5′-end of the effector protein gene *sipB* translationally fused to TEM beta-lactamase gene devoid of its region coding for the signal sequence [31]. We chose to use cultures propagated in cell culture medium as this is the medium used to generate invasion competent bacteria, and as the *lacZ* reporter activities did not differ between the wild type and mutant in this medium (Fig. 5C and D). When grown to generate invasiveness, we noted a clear presence of the SipB-Bla fusion protein in the culture supernatant and cell lysates from the wild type bacteria carrying pAUN1 as indicated by the enzymatic activity of the SipB-Bla fusion protein (Fig. 5E). For the *scsABCD* mutant we could likewise detect enzymatic activity from both fractions. However, as compared to the activities expressed by the wild type, the proportion of the extracellular activity was reduced and the cell-bound activity increased in the *scsABCD* mutant (Fig. 5E). The enzymatic activity became much reduced when the medium was supplemented with CuCl_2_ at 2 mM (Fig. 5F), and decreased below detection limit at 3 mM (data not shown). Interestingly though, at 2 mM CuCl_2_, the proportions of enzymatic activities for cell-bound and secreted fusion protein appeared the same for the wild type and *scsABCD* mutant (Fig. 5F).

To exclude any potential distractions emerging from alternations in specific enzyme activity generated by CuCl_2_, we also assayed for the SipB-Bla fusion protein by immunoblotting. In the absence of CuCl_2_, the amounts of the fusion protein in the supernatant appeared much higher in the wild type, and with a substantial accumulation in cell lysate for the Δ*scsABCD* mutant (Fig. 5G). At 2 mM CuCl_2_ the signals for the fusion protein decreased, but remained detectable upon longer exposure. Still, the proportions of the fusion protein in the extracellular versus intracellular fraction of the wild type appeared more pronounced compared to the Δ*scsABCD* mutant (Fig. 5G).

Combined, these observations indicated that while SPI1 expression was redox sensitive, the *scs* gene cluster did affect *prgH* expression in LB. Under conditions where the *scsABCD* deletion mutant did not reveal difference in *prgH* expression, the deletion still affected secretion of a SipB fusion protein.

### The *scsABCD* Genes, Intracellular Replication and *in vivo* Virulence of *S*. Typhimurium

**Figure 6:**
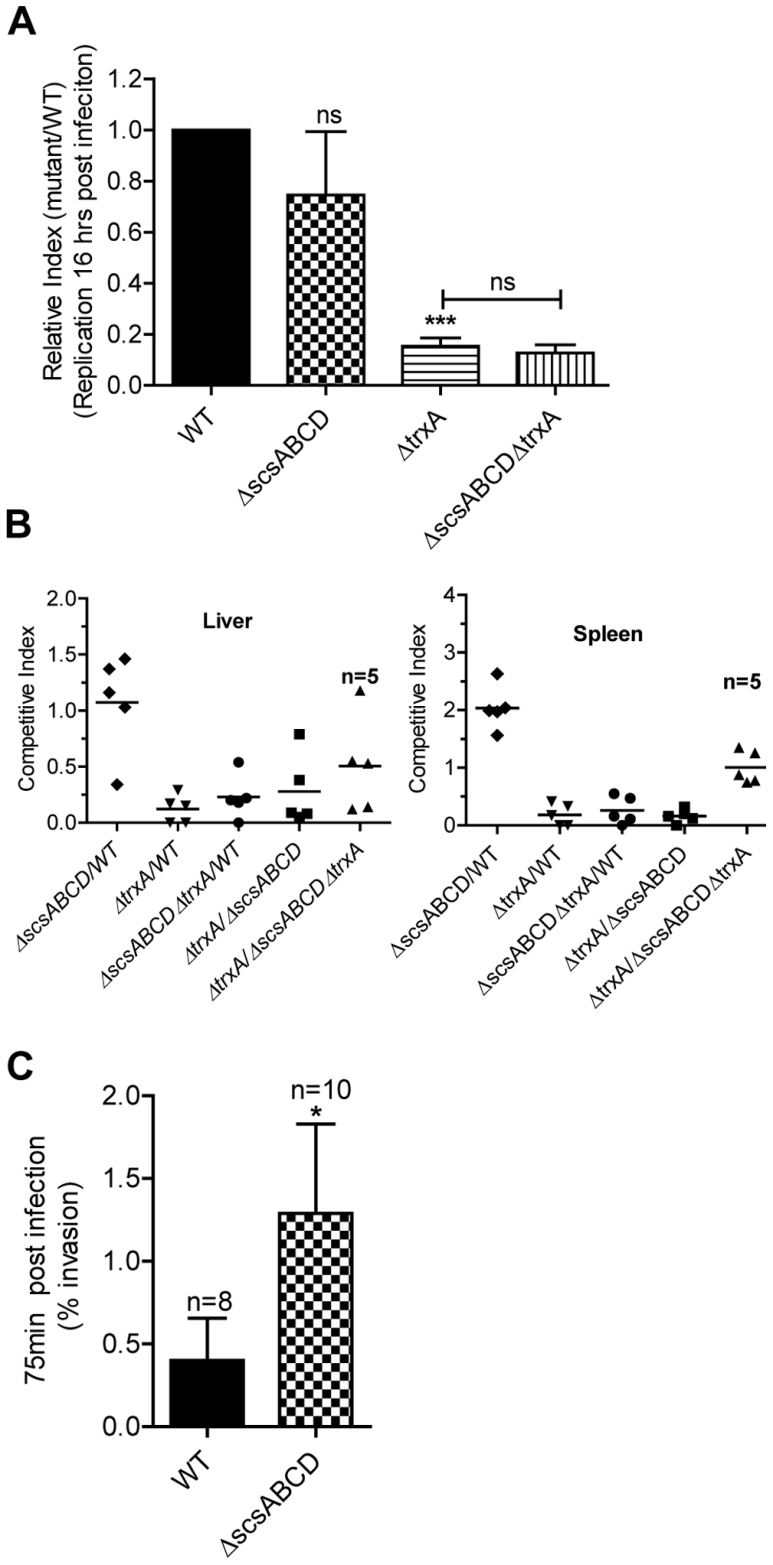
Replication in RAW264.7 macrophages and mice, and invasion in ileal loops of mice. A) The Δ*scsABCD* does not restore replication of Δ*trxA* in macrophage cell line RAW 264.7 B) The Δ*scsABCD* mutation shows a moderate increase in spleenic load in BALB/c mice on intraperitoneal challenge; n = number of mice C) The Δ*scsABCD* invades more efficiently in ileal loop ligation experiment. n = number of loops. ns = non-significant; * = p≤0.05; *** = p≤0.001. Error bars indicate standard error of the mean.

Apart from affecting invasion, TrxA strongly affects bacterial intracellular replication in RAW264.7 macrophage-like cells, as well as virulence in BALB/c mice [20]. Regarding intracellular replication in RAW264.7 cells we did not note any significant deviation for the Δ*scsABCD* mutant compared to the wild type (Fig. 6A).

Upon intraperitoneal challenge the Δ*scsABCD* mutant showed a moderate relative increase in spleenic loads three days post challenge (Fig. 6B). When comparing the ratios of wild type and mutant bacterial ratios by a nonparametric test, the difference did not breach statistical significance. However, we also extracted the actual viable counts of individual strains from the data to allow the use of a parametric test. In this, the differences in viable counts became statistically significant (P<0.05). Despite this enhancement in the replication of *scsABCD* mutant, the Δ*scsABCD* deletion neither restored the strong attenuation exhibited by the Δ*trxA* mutant background in RAW264.7 nor in mice (Figs. 6A and 6B). In other words, in contrast to the decreased invasiveness, the attenuation of the Δ*trxA* mutant in RAW264.7 cells and BALB/c mice evidently did not depend on the *scs* genes.

SPI1 is important in mediating invasion of *S.* Typhimurium from the intestine of mice. However, the redox conditions in the intestine are likely to deviate from the ones prevailing in cell culture settings. Hence we continued by comparing invasiveness of wild type and Δ*scsABCD* mutant of *S*. Typhimurium in ileal loops of BALB/c mice. In these experiments, the Δ*scsABCD* mutant indeed revealed an increase in numbers of invading bacteria (Fig. 6C).

### The *scsABCD* Gene Cluster Conditionally Suppresses Virulence in *C. elegans*

Feeding *C. elegans* with *S*. Typhimurium results in a significant shortening of the nematode life span, which in part relies on a massive hypodermal oxidative stress response in the nematode [22]. This stress response is initiated at the intestine some 24 hours post infection, and at 36 to 48 hours post infection fills the nematode hypodermal space, as revealed by staining with the fluorescent ROS indicator 2′,7′-dichlorodihydrofluorescein diacetate (H_2_DCFDA) [22]. Nematodes fed with a Δ*trxA* mutant of *S*. Typhimurium lack this oxidative response and gain a significant restoration of their life span [22]. Therefore, we set out to test whether *scsABCD* genes would bear any role in the pathogenesis of *S*. Typhimurium in *C. elegans*.

**Figure 7:**
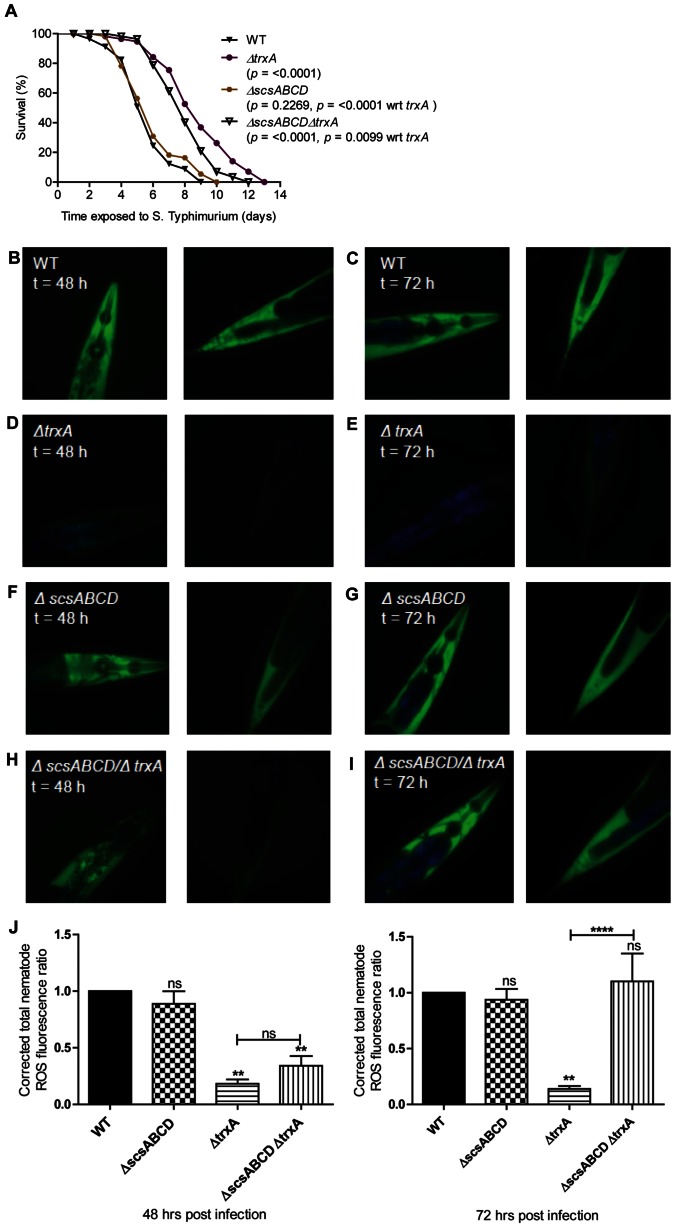
Virulence and ROS dissemination are restored in C. elegans by removing Δ*scsABCD* in the Δ*trxA* mutant. A) Survival of wild-type N2 nematodes was compared when infected with S. Typhimurium 14028 wild-type, Δ*trxA*, Δ*scsABCD* or the Δ*scsABCD*/Δ*trxA* mutant. B to I). Nematodes were harvested 48 or 72 hours post infection and stained with H_2_DCFDA to detect intracellular ROS. In these images, ROS is shown in green and intestinal autofluorescence in blue. Images are shown at 40x magnification and are representative of at least 20 nematodes from 2 independent assays. J) Florescence intensities are compared with wild type and between different mutants. ns = non-significant, ** = p≤0.01, **** = p≤0.0001. Error bars indicate the standard error of the mean.

*C. elegans* infected with the Δ*scsABCD* mutant of *S*. Typhimurium behaved as nematodes infected with the wild type regarding life span (Fig. 7A). Still, transfer of the *scsABCD* deletion into the Δ*trxA* mutant resulted in retrieved virulence as evidenced by a shortening of the nematode life span (Fig. 7A).

Nematodes infected with the wild type or Δ*scsABCD* mutant evoked an apparently equal hypodermal ROS staining with H_2_DCFDA (wild type, Figs 7B and C; Δ*scsABCD* mutant, Figs. 7F and G). This observation was corroborated by quantification of oxidative stress induced fluorescence of H_2_DCFDA (Fig. 7J). To corroborate our previous finding [22], the oxidative stress response was much reduced upon infection with the Δ*trxA* mutant (Figs. 7D and E, Fig. 7J). Surprisingly, infection with the Δ*scsABCD/*Δ*trxA* mutant accompanied with a substantial restoration in the hypodermal oxidative stress such that the H_2_DCFDA staining reached wild type levels at 72 h post infection (Figs. 7H and I, Fig. 7J).

Thereby, the *scsABCD* genes affected the Δ*trxA* mutant phenotype not only regarding invasiveness of epithelial cells, but also regarding the virulence and pathogenesis of the Δ*trxA* mutant in *C. elegans*.

## Discussion

Reactive oxygen species play an important role in the pathogenesis of many bacterial species both in cell culture setting and for virulence in man, mice and nematodes [12], [13], [14], [15], [16], [17]. In *E. coli* selected oxidoreductases of the thioredoxin superfamily, including thioredoxin 1 (TrxA) itself, are not only needed for tolerance to H_2_O_2_ but also for resistance to the redox active transition metal copper [30], [32], [33]. Copper ions mediate non-enzymatic catalysis of protein disuplhide formation [34], [35], which mimics a facet of oxidative stress that interferes with periplasmic disulphide bond formation. *S. enterica* carries a gene cluster, the *scsABCD* genes which, when expressed in copper sensitive laboratory strains of *E. coli,* enhances their copper tolerance [24]. Recently, copper was coupled to the ability of phagocytes to control infection with *S*. Typhimurium [36]. Furthermore, when *S*. Typhimurium replicates in macrophages, the *scs* genes are significantly induced for their expression [37]. Given the coupling between tolerance to oxidative stress and copper [30], [32], [33], we have searched for a role of the ScsABCD proteins, all putative members of the thioredoxin superfamily of oxidoreductases, for oxidative stress tolerance and virulence in *S*. Typhimurium.

Individual Δ*scsB,* Δ*scsC,* Δ*scsD* and Δ*trxA* mutants showed equal sensitization to copper chloride, and that deleting the Δ*scsABCD* genes in a Δ*trxA* mutant did not cause further sensitization. This deduced that the *scsABCD* and *trxA* genes contributed to copper tolerance through a common pathway. This conclusion would fit well with recent analyses in *C. cresentus* where ScsB represented a DsbD-like cytoplasmic membrane electron transporter and ScsC, a putative periplasmic disulphide isomerase and a reduction substrate for ScsB [25]. TrxA is also known to provide DsbD with electrons [38], which could adopt a connection between the ScsABCD system and TrxA in mediating copper tolerance. Exposure to H_2_O_2_ resulted in protein carbonylation both in the wild type *S*. Typhimurium and in the strain lacking the *scsABCD* genes. Still, the level of protein carbonylation was higher in the Δ*scsABCD* mutant as compared to wild type, notably in the periplasmic sub-cellular fraction. Such results further implicate a role of the Scs proteins in balancing redox stress in *S*. Typhimurium.

ScsA did not add to CuCl_2_ tolerance despite the presence of a *Cys-X-X-Cys* motif. However, this motif resides within its predicted signal sequence. Hence a processed ScsA would be missing a thioredoxin-like catalytic motif. Such a scenario invites for speculating that processing of ScsA could rely on disulfide formation in the putative signal sequence, and hence depend on periplasmic redox status for processing and transport. Still the Δ*scsA* mutant showed increased sensitization to H_2_O_2_, while no such sensitization was noted for any of the other *scs* deletion mutants or for the Δ*scsABCD* mutant. That is, an imbalance in the Scs protein content rather than lack of ScsA might explain the selective sensitization to H_2_O_2_. Such reports have already been published with periplasmic protein DsbA, where overexpression of DsbA protein suppresses the motility contrary to the requirement of DsbA for proper motility [39]. This said, we could not at present functionally distinguish ScsA from the rest of the Scs proteins or TrxA. The strong contribution of thiol peroxidase and methionine sulfoxide reductases for protection from H_2_O_2_ mediated killing in *S*. Typhimuirum [17] could further explain the redundancy of ScsBCD proteins in mediating any H_2_O_2_ stress tolerance *in vitro*.

When the Δ*scsABCD* mutant was tested in cell culture based virulence assays we did not note any alterations in its ability to invade epithelial cells or to replicate in macrophage-like cells. We did however observe that CuCl_2_ blocked invasion with extreme efficacy. Furthermore, this decrease in invasion strongly relied on the *scsABCD* and *trxA* genes. When given intraperitoneally to BALB/c mice, the Δ*scsABCD* mutant showed a moderate enhancement in fitness as compared to the wild type. Also, when assaying invasion in ileal loops of mice, we noted an enhanced invasion efficacy for the Δ*scsABCD* mutant.

Apart from exhibiting decreased invasiveness *in vitro*, a Δ*trxA* mutant of *S*. Typhimurium also reveals strong virulence attenuation in the soil nematode *C. elegans* [22]. Here we demonstrate that both these attenuations depended on the *scs* genes. That is, selected attenuations caused by CuCl_2_ or TrxA-deficiency are “conditionally” dependent on the *scs* genes.

Conditional phenotypes for oxidoreductase mutants of the thioredoxin superfamily have previously been described in *E. coli*. For example, H_2_O_2_ tolerance of *E. coli* relies on TrxA [30]. Surprisingly though a thioredoxin reductase (TrxB) deficient mutant while sensitive to H_2_O_2_ in stationary phase, shows even an increased tolerance to H_2_O_2_ in logarithmic phase of growth. This conditionality with H_2_O_2_ sensitivity comes from the fact that TrxB-deficiency associates with increased catalase expression evidently induced by accumulated oxidized TrxA [33]. A second example comes from the conditional contribution of oxidoreductases to copper chloride. In *E.coli*, lack of the periplasmic disulfide isomerase DsbC results in copper chloride sensitization, while deleting the disulfide oxidase DsbA does not [40]. Still, CuCl_2_ sensitivity is enhanced in strains lacking both DsbA and DsbC. The explanation to this apparent contradiction may come from CuCl_2_ itself; CuCl_2_ is an efficient but evidently a non-specific oxidant that introduces non-native disulfide bonds in periplasmic proteins such as alkaline phosphatase [40]. Thus in the absence of a proteins catalyzing and proof-reading disulphide bond formation, CuCl_2_ might cause accumulation of wrongly oxidized proteins to the extent of compromising bacterial fitness.

Assuming the Scs system participates in periplasmic redox shuffling, its conditional effects on virulence could relate to mechanisms similar to that proposed for the TrxA-TrxB and DsbA-DsbC interactors; presence of CuCl_2_ or TrxA-deficiency could cause differential Scs-mediated accumulation of oxidized periplasmic proteins. Such accumulating proteins, being the Scs proteins themselves or their substrates, could subsequently relate to altered T3SS activity, and thus explain the containment of the *scsABCD* genes in *S. enterica*. Indeed, the DsbA activity of *S.* Typhimurium has been connected to both SPI1 gene expression and in the SPI1 T3SS apparatus functionality [41]. Such connections led us to probe for the impact of the *scsABCD* gene cluster for SPI1 gene expression and secretion potential. When applying CuCl_2_ stress in cell culture medium, the transcriptional activity of the *hilA* and *prgH* promoters were reduced, as was the expression of the SipB-Bla fusion protein. Still, whether applying copper stress or not, the Δ*scsABCD* mutant appeared defective in secreting the SipB-Bla fusion protein. Clearly, the fusion protein does not represent an original T3SS effector protein, but the results still implicate a role for the Scs system in balancing SPI1 T3SS activity.

The above findings also provide a plausible mechanistic explanation for the invasiveness of the mutant. Under ordinary conditions the expression of SPI1 T3SS mediated invasion genes in wild type is highly active but leaky allowing secretion of effector proteins even in the absence of host cell contact. In the Δ*scsABCD* mutant the apparatus is less active, or stricter. Effector proteins accumulating inside mutant bacteria create a secretion competent pool applicable even after copper induced down-regulation of SPI1 gene expression, and notably, only used for translocation. Effectors secreted by SPI1 activate host JNK and p38 kinases, as well as nuclear localization of transcription factor NF-kB [9]. These kinases and transcription factors are described to potentiate expression of the inducible nitric oxide synthase [42], [43] as well as the phagocyte oxidase subunit gp91 [44]. Thereby a rational for *S. enterica* maintaining the *scs* genes could relate to the ability of regulating SPI1 secretion activity.

The redox-dependency of T3SS is not restricted to *S*. Typhimurium. The activity of the virulence-associated T3SS of *Pseudomonas aeruginosa* and *Shigella flexneri* relies on their DsbA homologues [45], [46]. Furthermore, expression of fimbrial adhesins in uropathogenic and enterotoxogenic *E. coli* [47], [48] as well as secretion of cholera toxin in *Vibrio choleare* [49] rely on corresponding DsbA homologues. Such reports in combination with our data on the *scs* genes implicate dependence and a highly balanced interaction of virulence factors with evolutionary conserved bacterial oxidoreductases belonging to the thioredoxin superfamily. Thereby, phenotypes caused by mutations in genes for oxidoreductases could be conditional in more general terms, and not restricted to *scs* genes in *S*. Typhimurium. If indeed so, then one must consider that mutating oxidoreducates would not only cause alterations in virulence but also act as suppressor mutations.

## Materials and Methods

### Ethical Statement

The work did not involve human subjects or non-human primates. However, the mice were used in this study and the ethical permits were obtained from Stockholm Norra djurförsöksetiska Nämd with N491/11 for intraperitoneal challange and N172/08 for ileal loop infections. The animals were housed and monitored according to the national and international guidelines.

### *In silico* Analyses

The *S.* Typhimurium strain LT2 and 14028s genome sequences annotated in 2001 [26] and 2010 [50] respectively, were used as reference for all the *in silico* analyses and primer design. Sequences from additional genome annotations were searched and compared in NCBI protein cluster for *Cys-X-X-Cys* motives. Search for Lipobox, classical signal peptide sequences and cleavage sites were conducted using the LipP 1.0 and SignalP servers [51], while protein localization and topologies were predicted using PSORTb vs3.0.2 and the Phyre2 server respectively [52].

### Bacterial Strains, Plasmids, Phages and Nematodes

*S.* Typhimurium 14028 (ATCC, Manassas, VA, USA) was used as the wild type *S*. Typhimurium strain throughout the study. The plasmids used for site-directed mutagenesis [29] were pDK3, pDK4 and pDK46. For cloning purposes, the pBAD derivative pBAD33, the pACYC-184 derivative pSU41 and T7 promoter based expression vector pET32a (Novagen) were used [53], [54], [55]. The primers for cloning are given in Table S1. Cultures were propagated in Luria broth or on Luria agar plates with 10g sodium chloride per liter (Duchefa Biochemie, The Netherlands). For generating invasive bacterial cultures, bacteria were propagated in defined cell culture medium as given below. When necessary, growth media were supplemented with ampicillin (100 µg/ml), chloramphenicol (10 µg/ml), kanamycin sulfate (50 µg/ml) or tetracycline (10 µg/ml). To activate the arabinose promoter in pBAD33, cultures were supplemented with L-arabinose to a final concentration of 1% (w/v). All antibiotics and L-arabinose were from Sigma-Aldrich (St. Louis, MO, USA). Phage P22 *int* transduction was used to transfer mutations between strains [56].

The *C. elegans* strain used in this study was wild-type variant Bristol N2. Nematodes were cultured and maintained at 20°C on modified nematode growth media (NGM, 0.35% peptone) agar plates and fed with *E. coli* strain OP50, as described [57]. Both strains were obtained from the *Caenorhabditis* Genetics Center (Minneapolis, USA).

Bacterial strains and plasmids used and generated in this study are summarized in Table 1.

**Table 1:** Strains and plasmid.

Strains	Genotype/Property	Reference
MC5	Wild Type	ATCC 14028
Fia-1280	Wildtype::Tet^r^	[63]
NA48	14028(Δ*scsA)*	This study
NA49	14028(Δ*scsB)*	This study
NA50	14028(Δ*scsC)*	This study
NA51	14028(Δ*scsD)*	This study
NA91	14028(*ΔscsABCD)*	This study
NA198	14028(*ΔscsABCD/ΔtrxA*)	This study
Fia-1195	14028(*ΔtrxA*)	[20]
Fia-1381	14028(*ΔprgH*::Tn5 *lacZY*)	[63]
Fia-1199	14028(*ΔhilA*::Tn5 *lacZY*)	[63]
**Plasmids**		
pSU41	pACYC-184 derivative vector control, Kan^r^	[54]
pNA10	The *scsABCD* gene cluster cloned in pSU41 with 209 bp upstream region of *scsA*, Kan^r^	This study
pAUN1	*sipB*-β-lactamase fusion cloned in pSU41, Kan^r^	[64]
pET32a	T7 promoter based expression vector	[55]
pNA14	The *scsA* cloned in BamHI and XhoI site of pET32a, Amp^r^	This sutdy
pNA15	The *scsB* cloned in EcoRI and XhoI site of pET32a, Amp^r^	This sutdy
pNA16	The *scsC* cloned in BamHI and XhoI site of pET32a, Amp^r^	This sutdy
pNA17	The *scsD* cloned in BamHI and XhoI site of pET32a, Amp^r^	This sutdy
pBAD33	pBAD series vector control, Cm^r^	[53]
pFA3	Full length *trxA* cloned in pBAD33, Cm^r^	[20]
pFA8	Catalytically inactive *trxA* cloned in pBAD33, Cm^r^	[20]

### Generation of Knockout Mutants

Individual *scs* genes, or the entire *scsABCD* locus, were deleted in the wild type *S*. Typhimurium using phage recombinase-assisted homologous recombination [29]. Briefly, the resistance genes from pDK3 or pDK4 were PCR-amplified with primer extensions homologous to individual *scs* genes, as defined in Table S2. PCR products were electroporated into the recipients containing pDK46 and recombinants selected using proper antibiotic selection. To exclude possible secondary mutations, the inserted cassettes were transduced by the phage P22 *int* into a fresh *S*. Typhimuirum 14028 background. Finally, the antibiotic resistance cassettes were removed with the aid of plasmid pCP20 coding for recombinase [29].

All mutants were verified by PCR amplification of inserted resistance cassette with primers designed 100 bp up- and downstream of the target genes respectively, as listed in Table S3.

### Protein Expression and Cell Fractionation

Individual *scs* genes with C-terminal His-tags were cloned into the cloning site of pET32a expression vector. The clones were transformed to *E.coli* BL21 strain. The cultures were grown overnight in 2 ml LB and were subcultured as 1∶100 in 20 ml of LB with aeration at 37°C. The cultures were either induced with 2 mM IPTG or left uninduced and samples were collected after 3 hrs. Harvested cultures were fractionated into cytoplasmic, Triton X-100 (Tx-100) soluble, Tx-100 insoluble and periplasmic proteins as described in [58], [59] and [60] respectively.

For generatio of Tx-100 soluble, Tx-100 insoluble fractions, the bacteria were pelleted and the pellets were resuspended in PBS on ice. Chilled suspensions were sonicated in 4 ml volume using the Soniprep 150 SANYO at 12 micron amplitude for 2×1.5 min on ice. Whole Cells were removed by centrifugation at 5000 rmp for 10 min. Supernatants were collected and centrifuged for 15 min at 14000 rpm and 4°C. The supernatant was collected as cytoplasmic soluble fraction. The pellet was solubilized in cytoplasmic membrane solution; 50 mM Tris-HCl, 10 mM MgCl2, 1% (v/v) Triton X-100 buffer (pH 8.0), and centrifuged aforesaid. The supernatant containing cytoplasmic membranes was collectd as the TX-100 soluble fraction. The pellet containing integral outer membrane proteins was solubilized in reducing SDS sample buffer.

For periplasmic proteins, the pellet from 5 ml culture was resuspended in 50 µl of chloroform, vortexed and kept at room temperature (22°C) for 15 minutes. Five hundred microlitre of 0.01 M Tris hydrochloride (pH = 8.0) was added and mixed. After centrifugation at 6000xg for 20 minutes, the aqueous supernatant containing periplasmic proteins was collected [60].

Fractions were separated using SDS-polyacrylamid gel electrophoresis [61] and transferred to PVDF membrane by using iblot system (invitrogen) The proteins were detected by using HRP conjugated monoclonal anti-His antibody and ECL substrate (SuperSignal West Pico Chemiluminescent Substrate, Thermo Scientific). Biorad Gel doc machine was used for signal capture.

For mass-spectrometry, protein bands were cut from the gel, washed with water and reduced by DTE and alkylated with iodoacetamide. Proteins were digested with trypsin (Promega) in 50 mM NH_4_HCO_3_ at 37°C overnight. Extracted peptides were analyzed with MALDI-Tof analysis using a Bruker Ultraflex-Tof/Tof instrument. Identification was done by using the peptide maps for search in the NCBInr database using the Mascot search engine.

### Copper Chloride, Zinc Chloride and Hydrogen Peroxide Tolerance

Bacteria were grown overnight on Luria agar (LA) plates and re-suspended in phosphate buffered saline (PBS, pH = 7.4). For testing sensitivity to either CuCl_2_ or ZnCl_2_ (Sigma Aldrich), bacteria were inoculated from PBS into 2 ml of LB to contain 10^3^ bacteria per ml. Cultures were supplemented with CuCl_2_ to a final concentration of 0 mM, 2 mM, 4 mM or 6 mM. For ZnCl_2,_ the final concentration was adjusted to 0 mM, 0.25 mM, 0.5 mM, 0.75 mM, 1.0 mM, 1.25 mM, 1.5 mM, 1.75 mM and 2.0 mM respectively.Tubes were incubated at 37°C overnight on Brunswick roller. Overnight cultures were diluted 1∶10 in PBS and OD_600_ were recorded, taking respective reaction mixture as blank. For H_2_O_2_ test_,_ 10^3^ bacteria in 100 µl of PBS were mixed with 100 µl of H_2_O_2_ (Sigma) solution in 96-well microtitre plate, in triplicate, giving final concentration of H_2_O_2_ as 0 mM, 1 mM, 2 mM and 4 mM in PBS respectively. Plates were incubated at 37°C for 2 hours and 100 µl from each well was spread on LA plate. The seeded plates were incubated overnight at 37°C. Viable counts were determined by counting CFU on plates and percent viability as related to wells devoid of H_2_O_2_ were calculated.

### Detection of Protein Carbonylation

The protocol detailed by [17] was followed with slight modifications. Overnight 2 ml LB cultures of *S.* Typhimurium were diluted 1∶100 in fresh 25 ml LB and grown for 3.5 hrs at 37°C at 200 rpm. Cultures were exposed to H_2_O_2_ at a final concentration of 0.75 mM and incubated for further 3 hours at 37°C without shaking. Cells were then centrifuged at 5000 rpm and pellet was resuspended in 1 ml of 10 mM Tris-HCl, 25 mM NaCl and 50 mM DTT (pH 7.5) and transferred to 2 ml Eppendorf tube. Cells were mechanically lysed using the Soniprep 150 SANYO at 20 micron amplitude for 3 min on ice. The cell debris was removed by centrifugation at 14000 rpm for 10 minutes. After another centrifugation step, the clear supernatant was collected.

The periplasmic proteins were isolated according to [60] and as described above.

Oxidized proteins content was detected by following the manual provided with OxyBlot™ protein oxidation detection kit (Millipore). Briefly, 5 µl from each culture supernatant was mixed with 5 µl of 12% SDS. After 5 min 10 µl of the derivatizing reagent 1 x DNPH (dinitrophenyl hydrazine) was added and incubated at room temperature (22°C) for 15 min. Reaction was stopped by adding 7.5 µl neutralization solution provided in the kit. The samples were analyzed on a 12% SDS-PAGE.

The proteins were blotted onto a PVDF membrane (Amersham Hybond™-P, GE Healthcare), and subsequently were blocked overnight in blocking buffer (PBS with 1% BSA and 0.05% Tween 20). The membrane was incubated in rabbit anti-DNP-antibody (1∶150), diluted in blocking buffer, for 1 hr at room temperature with shaking. After subsequent washing with PBS-T (0.05%) goat anti-rabbit IgG horseradish peroxidase conjugate (1∶300), diluted in blocking buffer, was applied for 1h. Oxidized proteins were detected by using the ECL substrate (SuperSignal West Pico Chemiluminescent Substrate, Thermo Scientific) and Biorad Gel doc machine.

### *In vitro* Invasion Assays

To generate invasive cultures of *S*. Typhimrium 14028, overnight cultures propagated in Luria broth were diluted (1∶10) in 4 ml D-MEM (Gibco) supplemented with 10 mM HEPES and 10 mM L-Glutamine as described in [20]. The cultures were grown in 15 ml polystyrene plastic tubes under shaking for 4 hours lacking or being exposed to 3 mM CuCl_2_. MDCK epithelial cell line was maintained and infected as described pereviously [20]. Briefly, the confluent cells in 24 well plates were exposed to 1×10^6^ bacteria (MOI 10∶1) diluted from the invasion competent culture in 1 ml buffered D-MEM devoid of serum and gentamicin. Invasion was synchronized by briefly centrifuging the plates, where after plates were incubated for 1 hour at 37°C. After washing the monolayers twice with PBS, cells were covered with 10%FBS supplemented D-MEM containing 50 µg/ml of gentamicin for 45 minutes. Subsequently, the cells were washed twice with PBS and bacteria were released by lyzing cells in 0.5% sodium deoxycholate in PBS (Merck, Darmstadt, Germany). Invasion efficacy was defined as the ratio of recovered CFU in relation to the bacteria enumerated from the inoculum.

### Promotor-fusions and β-galactosidase Measurements

The transcriptional activities for *hilA::lacZY* and *prgH::lacZY* promotor fusions were determined as described previously [62] from overnight cultures in LB, or from bacteria grown to induce invasiveness in cell culture medium. The *hilA::lacZY* and *prgH::lacZY* constructs, received from Catherine A. Lee [63], were transduced in *scsABCD* mutant and wild type 14028 background.

### SipB-β-lactamase Fusion Proteins Determination

Activity of the fusion protein was assayed either enzymatically or by immunoblotting. For determining the β-lactamase activities, pAUN1 plasmid carrying sipB-β-lactamase fusions were transformed in respective strains and enzymatic activities from the supernatants and whole cell lysates were determined as described by Negrea *et al.* [64]. Briefly, the invasive cultures, optically normalized, were centrifuged at 5000 rpm to collect the clear supernatant and the cells pelleted. Pellets were resuspended in PBS and were sonicated as described above. The proteins from the supernatant as well as from cell lysate were precipitated with 10% final concentration of trichloro acetic acid and resuspended in reducing SDS sample buffer. Equal volumes of protein preparations were run on SDS-polyacryamide gel and blotted to PVDF membrane with iblot-system (Invitrogen). The proteins were detected by using Rabbit-anit-β-lactamase antibody (Abcam) in 1∶3000 dilutions. After treating with anti-rabbit HRP-conjugated secondary antibody, the bands were detected as described earlier.

### Intracellular Replication in RAW264.7 Cells

Murine macrophage-like RAW264.7 cells were grown in RPMI (Gibco) supplemented with FBS, HEPES, L-glutamine and gentamicin as described by [20], [65]. To assay for possible alterations in intracellular replication, bacteria were diluted from overnight plate cultures in PBS, opsonized with mouse serum (10% v/v final concentration) for 30 minutes, and diluted in supplemented RPMI (Gibco) devoid of serum and gentamicin to represent 1×10^6^ bacteria per ml. The macrophages were exposed to 1 ml of inoculum in 24-well plates. To synchronize phagocytosis, plates were briefly centrifuged and incubated at 37°C for 1 hr. Cells where after washed twice with PBS and exposed to serum supplemented RPMI containing 50 µg/ml of gentamicin for 45 minutes. At this point, a 1 hr sample was collected after washing the cells in PBS and releasing bacteria by hypotonic lysis of cells. The second sample was collected at 16 hrs post infection. Growth yields were defined as the ratio in viable counts from the 16 and one-hour samples.

### Infection Experiments in Mice

Female BALB/c J mice at the age of 6 to 8 weeks were purchased from Taconic Europe (Denmark) and housed at Microbiology Tumor and Cell biology (MTC) animal facility, Karolinska Institutet, Stockholm, Sweden, under normal conditions in accordance with both institutional and national guidelines. For competition experiment, each group of mice (n = 5) was infected intra-peritoneal by 1×10^4^ bacteria/100 µl of infection dose consisting of wild-type, tagged with a tetracycline resistance marker inserted at a neutral genomic position, and mutant mixture in 1∶1 ratio [66]. The mice were sacrificed on third day post infection and livers and spleens were excised, homogenized in PBS and appropriate dilutions were placed on L-agar plates. Both members in each infection mixture were separated by appropriate resistance conferred by each of them [67]. Competitive indices (CI) were calculated as described previously [68].

The ileal loop infection experiment was carried out as described earlier [69]. The mice were deprived of water 4 hrs prior to the experiment. Mice were anaesthetized with isofluorane and were operated to expose the bowl. The ileum was located and near cecum, minimum 1.5 cm long piece was ligated at both ends taking care not to cut the ileum. 10^8^ bacteria in a dose of 100 µl were injected into the ligated part. The bowl of the mouse was stitched aseptically and mice were allowed to wake up. The mice were closely observed for 75 min and then sacrificed by cervical dislocation. The bowl was re-opened and ligated loop was cut apart and taken out. The loops were cut open, washed in PBS and extracellular bacteria were killed with the application of gentamicin at 100 µg/ml in PBS for 1 hr. The washed ileum tissue was smashed in PBS and appropriate dilutions were plated to get the viable counts.

### Infection Experiments in *C. elegans*

Bacterial strains were grown overnight in LB broth at 37°C and lawns were prepared by spreading 200 µl of overnight culture on modified NGM agar. 20 L4-staged wild-type N2 *C. elegans* were subsequently infected as described [70]. Briefly, nematodes were set down on unseeded agar before transferring to bacterial lawns to reduce, as much as possible, the transfer of *E. coli*. Nematode survival was scored at 24°C and nematodes were considered dead when not responding to gentle touch by a platinum wire. Nematodes were also transferred to fresh bacterial lawns every day. Results are representative of triplicates from 3 independent assays.

### Detection of Reactive Oxygen Species in *C. elegans*

2′,7′-dichlorodihydrofluorescein diacetate (H_2_DCFDA; Sigma-Aldrich) was used to visualize intracellular ROS in nematodes. Stock aliquots of H2DCFDA (2 mM) were prepared in dimethyl sulfoxide and stored in the dark at −80°C. L4-staged N2 nematodes were infected as described above. At each time point, infected nematodes were harvested into tubes and washed twice with M9 buffer [57]. Nematodes were subsequently incubated with 25 µM H_2_DCFDA in 250 µl M9 buffer, in the dark for 30 min in a 20°C water bath. Nematodes were subsequently washed thrice with M9 buffer and mounted for microscopy in PBS with 25 mM sodium azide (NaN_3,_ Merck). Slides were visualized on a LEICA DMRE microscope and images were analyzed by GNU Image Manipulation Program. Total nematode ROS fluorescence intensities were quantified using ImageJ software and expressed as a ratio relative to wild-type ATCC 14028 as described previously [71]. Images are representative of at least 20 nematodes from 2 independent assays.

### Statistical Analyses

All the experiments were repeated at least three times and data were analyzed using the PRISM (version 5.0) software. The data for Figs. 2A to 2C were analyzed by linear regression and Fig. 2D further analyzed by t–test on the raw percent survival values. The data for Fig. 4, Fig. 5, Fig. 6A and Fig. 6C were analyzed by t-test. Data for Fig. 6B were analyzed by Wilcoxin signed-rank test. Fig. 7A data were analyzed by Kaplan–Meier estimation and Fig. 7J data were analyzed by Wilcoxin signed-rank test.

## Supporting Information

Table S1 **Primer sequences for cloning of** ***scs*** **genes.** *S.* Typhimurium LT2 genome sequence was used as reference for designing of all the primers for cloning.
(DOC)

Table S2 **Primers used for mutagenesis of** ***scs*** **genes.** Homologous overhangs in the mutagenesis primers are designed by taking *S.* Typhimurium LT2 genome sequence as reference.
(DOC)

Table S3 **PCR verification primers for mutants.** The primers were designed 100 bp up- and downstream of ORFs of *scs* genes to amplify the inserted antibiotic cassette. The reference genome sequence for primer designing was of *S.* Typhimurium LT2 strain.
(DOC)
