# Supplementary material for: Oxidoreductases that Act as Conditional Virulence Suppressors in Salmonella enterica Serovar Typhimurium
Source: PLoS One. 2013 Jun 4;8(6):e64948. doi: 10.1371/journal.pone.0064948 (PMC3672137; doi:10.1371/journal.pone.0064948)
Supplement: Table S1 — Primer sequences for cloning of scs genes. S. Typhimurium LT2 genome sequence was used as reference for designing of all the primers for cloning. (DOC) [file pone.0064948.s001.doc]

**Table S1**

| **Primers for cloning of *scs* genes** | | |
| --- | --- | --- |
| Plasmid | Sequence *(5´ – 3´)* | Target plasmid |
| pNA10 | Fw:gctctagagcCAATACATACAGAAGGGAC | The *scsABCD* genes cloned between XbaI and SacI site of pSU41 |
|  | Rv:CGAGCTCGGCGGATGACAACAAAACAG |  |
| pNA14 | Fw:CGGATCCGCTCGCGTTAAACAGTGAG | The *scsA* gene cloned between BamHI and XhoI site of pET32a |
|  | Rv:CCGCTCGAGTCAGTGGTGGTGATGATGATGTTCACGGAAGACACAAAA |  |
| pNA15 | Fw:GGAATTCCAGGAGGAACAGCTATGATGATTTTGTTCAGGCG | The *scsB* gene cloned between EcoRI and XhoI site of pET32a |
|  | Rv:CCGCTCGAGTCAGTGGTGGTGATGATGATGTTGGGTTATTCCTTTCGC |  |
| pNA16 | Fw:CGGATCCGCGATGCGGTATTACAAACG | The *scsC* gene cloned between BamHI and XhoI site of pET32a |
|  | Rv:CCGCTCGAGTTAGTGGTGGTGATGATGATGCCCGCCATTGGCAGACGC |  |
| pNA17 | Fw:CGGATCCGAGGAGGAACAGCCAATGGCGGGTAAACTG | The *scsD* gene cloned between BamHI and XhoI site of pET32a |
|  | Rv:CCGCTCGAGTCAGTGGTGGTGGTGGTGGTGGAACGTTTTTGCCCACCA |  |

*Fw = Forward primer; Rv = Reverse primer*
