# Supplementary material for: Oxidoreductases that Act as Conditional Virulence Suppressors in Salmonella enterica Serovar Typhimurium
Source: PLoS One. 2013 Jun 4;8(6):e64948. doi: 10.1371/journal.pone.0064948 (PMC3672137; doi:10.1371/journal.pone.0064948)
Supplement: Table S2 — Primers used for mutagenesis of scs genes. Homologous overhangs in the mutagenesis primers are designed by taking S. Typhimurium LT2 genome sequence as reference. (DOC) [file pone.0064948.s002.doc]

**Table S2**

| **Primers used for mutagenesis** | | | |
| --- | --- | --- | --- |
| Primer | Sequence *(5´ – 3´)****^*^*** | Target gene | Reference |
| F*scsA*rec | CCAGTGGCTAAGATAACTCGCGTTAAACAGTGA  GGGCGCAGTGTAGGCTGGAGCTGCTTC | *scsA* | STM1113 |
| R*scsA*rec | TCCGTGAATGAGTAATTAACCGTTAGCAATAACC  GGTCTGCATATGAATATCCTCCTTAG |
| F*scsB*rec | ATGATGATTTTGTTCAGGCGGATACTGTTCTGCCTGTTATGTGTAGGCTGGAGCTGCTTC | *scsB* | STM1114 |
| R*scsB*rec | TGCACATCCTCTTTTTGCAGGACGTTGTATTTATTCACTTCATATGAATATCCTCCTTAG |
| F*scsC*rec | CGGTATTACAAACGTTGAAAAAAGCGAAAGGAAT  AACCCAGTGTAGGCTGGAGCTGCTTC | *scsC* | STM1115 |
| R*scsC*rec | CGCCAGAAAAACCGCGGCTTCACGCAGCCAACG  CCGCAGTCATATGAATATCCTCCTTAG |
| F*scsD*rec | ACGCTGGAAGCGGTGGTGAAAGAAAAACTGGCG  TCTGCCAGTGTAGGCTGGAGCTGCTTC | *scsD* | STM1116 |
| R*scsD*rec | TACAGTAAACGCGTTAGCGCCGGGAAACCCCGG  CGCATATCATATGAATATCCTCCTTAG |
| ***^*^****Underlined sequences are homologous to template plasmids; pKD3 or pKD4* | | | |
