# Supplementary material for: Oxidoreductases that Act as Conditional Virulence Suppressors in Salmonella enterica Serovar Typhimurium
Source: PLoS One. 2013 Jun 4;8(6):e64948. doi: 10.1371/journal.pone.0064948 (PMC3672137; doi:10.1371/journal.pone.0064948)
Supplement: Table S3 — PCR verification primers for mutants. The primers were designed 100 bp up- and downstream of ORFs of scs genes to amplify the inserted antibiotic cassette. The reference genome sequence for primer designing was of S. Typhimurium LT2 strain. (DOC) [file pone.0064948.s003.doc]

**Table S3**

| **Primers for PCR verification of mutants** | | | |
| --- | --- | --- | --- |
| Primer | | Sequence *(5´ – 3´)* | |
| *scsA*EXTF | ATCAGAAGGTTGCGCAGC | |  |
| *scsA*EXTR | GCAGCCAAAGCCATAACA | |  |
| *scsB*EXTF | TTCGCCGCCCACATTACGGGT | |  |
| *scsB*EXTR | CAATCTGCTTTTCCTGATCCGGCGTA | |  |
| *scsC*EXTF | AGGTATATGGCCCCGGCT | |  |
| *scsC*EXTR | CCAGACGTCCATGACCACCAT | |  |
| *scsD*EXTF | CAAGGAACGCCAGCGACG | |  |
| *scsD*EXTR | TGAGTGTGACAGAAATGG | |  |
